# Vanadia–Zirconia and Vanadia–Hafnia Catalysts for Utilization of Volatile Organic Compound Emissions

**DOI:** 10.3390/ma14185265

**Published:** 2021-09-13

**Authors:** Satu Ojala, Tiina Laitinen, Sian Leneuf de Neufville, Mari Honkanen, Minnamari Vippola, Mika Huuhtanen, Riitta L. Keiski

**Affiliations:** 1 Environmental and Chemical Engineering, Faculty of Technology, University of Oulu, 90570 Oulu, Finland; tiina.laitinen@oulu.fi (T.L.); mika.huuhtanen@oulu.fi (M.H.); riitta.keiski@oulu.fi (R.L.K.); 2 Institut Universitaire de Technologie de Poitiers, Universite de Poitiers, 86000 Poitiers, France; sianleneuf@gmail.com; 3 Tampere Microscopy Center, Tampere University, 33100 Tampere, Finland; mari.honkanen@tuni.fi (M.H.); minnamari.vippola@tuni.fi (M.V.)

**Keywords:** environmental catalysis, utilization of VOC, time-gated Raman spectroscopy, poisoning, characterization, sol–gel method, impregnation

## Abstract

Utilization is a sustainable and interesting alternative for the destructive treatment of volatile organic compounds due to avoided CO_2_ emission. This work concentrates on the development of active and sulfur-tolerant catalysts for the utilization of contaminated methanol. Impregnated and sol–gel prepared vanadia–zirconia and vanadia–hafnia catalysts were thoroughly characterized by N_2_ sorption, analytical (S)TEM, elemental analysis, XRD and Raman spectroscopy, and their performances were evaluated in formaldehyde production from methanol and methanethiol mixture. The results showed higher activity of the sol–gel prepared catalysts due to formation of mono- and polymeric vanadia species. Unfortunately, the most active vanadia sites were deactivated more easily than the metal-mixed oxide HfV_2_O_7_ and ZrV_2_O_7_ phases, as well as crystalline V_2_O_5_ observed in the impregnated catalysts. Metal-mixed oxide phases were formed in impregnated catalysts through formation of defects in HfO_2_ and ZrO_2_ structure during calcination at 600 °C, which was evidenced by Raman spectroscopy. The sol–gel prepared vanadia–zirconia and vanadia–hafnia catalysts were able to produce formaldehyde from contaminated methanol with high selectivity at temperature around 400 °C, while impregnated catalysts required 50–100 °C higher temperatures.

## 1. Introduction

Emissions of volatile organic compounds (VOCs) represent an interesting possibility to be used in the production of valuable chemicals [1]. Especially when emitted from industrial sources, the quality and quantity of the emissions approach the characteristics where utilization could become economically feasible [1]. A variety of chemicals can be produced applying carefully designed catalytic materials, and at the same time carbon of the emission is retained in the product instead of its release to the environment. In general, utilization of the gaseous emissions could improve the overall sustainability of the production.

Apart from CO_2_, the utilization of gaseous emissions is a significantly less studied topic than recycling and use of the solid waste. Some examples exist in energy production and utilization of VOCs in H_2_ production [2]. One attractive possibility is to convert contaminated methanol emissions from pulp industry into formaldehyde, which was first proposed by Wachs [3]. The sulfur compounds coexisting with methanol in emission stream cause the well-known challenges to the catalytic process, and therefore catalytic materials with high resistance against sulfur-poisoning needs to be developed.

Earlier studies have demonstrated the activity of cobalt–alumina and cobalt–alumina-ceria catalysts [4], as well as vanadia supported on silica and titania [5] in formaldehyde production from contaminated methanol. Our earlier study has also demonstrated better performance of ZrO_2_ and HfO_2_ compared to Al_2_O_3_ support in the case of vanadia catalysts [6]. The better stability of the ZrO_2_ and HfO_2_ supported catalysts was proposed to originate from higher VO_x_ surface density, which led to formation of mixed oxide structures between vanadia and the support.

Hafnium and zirconium are tetravalent transition metals that have remarkably similar chemical properties. In nature, hafnium is always present together with zirconium [7]. Hafnium and zirconium are corrosion resistant metals, and they are stable against acidic compounds [7,8]. In catalysis applications, hafnium and zirconium are used in oxidic forms. As pure oxides, HfO_2_ and ZrO_2_ have three thermodynamically stable crystalline phases at ambient pressure: monoclinic (low temperature), tetragonal, and cubic phases [9]. HfO_2_ has also reported to have a fluorite and orthorhombic structure [10]. HfO_2_ (~2760 °C) and ZrO_2_ (2715 °C) have high melting points, giving them particularly good resistance to temperature. As an oxide, HfO_2_ is slightly more basic than ZrO_2_ [11]. ZrO_2_ is widely used in three-way catalytic converters as a solid solution with CeO_2_. In this application, ZrO_2_ is used to improve oxygen storage capacity and thermal stability of the catalyst [12]. HfO_2_ is less studied as a catalytic material. It finds the most common applications in electronic devices and refractory materials [13]. Both these oxides are interesting support materials for vanadia in the oxidation of contaminated methanol, since the reaction mixture contains acidic sulfur contaminants.

Vanadium is a very unusual catalytic material, since it can exist in several oxide forms depending on the surrounding conditions. In addition to principal oxides V_2_O_5_, VO_2_, V_2_O_3_, and VO, it can take several other oxide phases between these principal oxides and non-stoichiometric phases. When impregnated on the oxidic supports the nature of the vanadium species is dependent on the surface coverage. Below the monolayer, vanadia takes the forms of surface vanadium oxide species, while above the monolayer, V_2_O_5_ particles appear. The vanadia species on metal oxides are also dependent on the hydrophilic/hydrophobic nature of the support and the pH_PZC_ of the system defining the pH of the liquid film on the support in hydrated conditions. Since pH_PZC_ of V_2_O_5_ (~1.5) is typically lower than those of oxide supports (about 7.4 for ZrO_2_ [14] and 5–7.4 for HfO_2_ [14,15]), the overall pH_PZC_ decreases with higher vanadia loading. In the liquid film on the hydrophilic oxide support, vanadia polymerizes continuously when pH is decreased, starting from VO_4_^3−^ species and finally leading to V_2_O_5_ precipitate. Since the prepared catalysts are exposed to ambient moisture, it has been concluded that the surface species of the hydrated vanadium cannot be modified using different precursors or the preparation method of a catalyst, but it takes the form according to pH of the liquid film [16]. The hydration/dehydration of the supported vanadia is reversible. It means that the catalyst is dehydrated when used in dry conditions at higher temperature and hydrated when stored at ambient conditions before/after use. The dehydrated surface vanadia species are mainly present in +V oxidation state as VO_4_ species having one terminal V = O bond. Both oligomeric and isolated species exist on the surface, and the number of oligomeric species increases with increase of surface coverage [16]. Furthermore, while connection of low melting point of V_2_O_5_ (690 °C) and thus low Tamman temperature (~200 °C) and differences between surface free energy of V_2_O_5_ and oxidic support explains why formation of vanadia layer on oxide support appears before formation of V_2_O_5_ crystals [16]. It also indicates the mobility of surface vanadia at the temperatures typical for the VOC utilization (350–450 °C) [5,6]. This may lead to changes in catalyst performance during their use.

The aim of the current study was to achieve more information on vanadia catalysts supported on hafnium and zirconium oxides. The catalysts were made by wet impregnation and sol–gel methods to discover possibilities to stabilize vanadia in the structure of the oxides. In this regard, we aimed to study the formation of metal-mixed oxide phases between vanadia and the support in different cases to better explain the phenomena observed earlier related to the poisoning and the selectivity of the catalysts.

## 2. Materials and Methods

### 2.1. Preparation of the Catalysts

The vanadia–zirconia and vanadia–hafnia catalysts were prepared by wet impregnation and sol–gel methods. In connection with the wet impregnation, commercial ZrO_2_ (99%, 5 μm, Sigma-Aldrich, St. Louis, MO, USA) and HfO_2_ (99%, −325 mesh ~<44 μm, Alfa Aesar, Haverhill, MA, USA) supports were first calcined at 600 °C for 4 h. Calculated amounts of vanadyl acetylacetonate VO (acac)_2_ (98% Sigma-Aldrich) were dissolved in methanol (99.9% Merck, Darmstadt, Germany) and mixed with the calcined supports. Wet impregnation was performed at room temperature for 20 h. After impregnation, the samples were dried at first on a sand bath at 90 °C for 5 h and then in a heated oven at 120 °C overnight. Finally, the samples were calcined at 600 °C for 4 h (catalysts denoted as VZr Imp and VHf Imp). To compare the effect of calcination temperature and time, VZr was calcined also at 500 °C for 2 h (catalyst denoted as VZr Imp 500 °C) and 4 h (information related to this catalyst is given in supplementary information).

Preparation method of vanadia–zirconia by the sol–gel method was modified from the information published in [17,18]. The major difference in the used preparation comes from different vanadium precursor. The sol–gel method is known to be rather simple and produce homogeneous materials. At first, zirconium (IV) oxynitrate hydrate was dissolved in milliQ water and stirred at room temperature for 45 min. Then, VO (acac)_2_ dissolved in methanol was added into zirconium solution and the temperature was raised to 60 °C. After 1 h stirring, citric acid was added to the solution in molar ratio of acid to metal (Zr + V) 2:1. When acid was completely dissolved, the pH of the solution was adjusted to 10 by using ammonia. After the addition of ammonia, the color of the solution changed to dark blue. Stirring was continued at 60 °C for 20 h. The solution was dried gently on the sand bath at 70 °C for 24 h and then in the oven at 120 °C for 24 h. Finally, the calcination of sample was performed at 600 °C for 4 h (catalyst denoted as VZr SG).

Preparation of vanadia–hafnia by the sol–gel method was developed based on the literature [19,20]. As a difference to the references, dissolved vanadium precursor was added in the hafnium chloride solution. At first, hafnium chloride (HfCl_4_) was dissolved in ethanol and stirred at the room temperature for 30 min. Then, VO (acac)_2_ dissolved in ethanol and milliQ water was added into hafnium solution. The molar ratio of HfCl_4_ to water was 1:4. Stirring was continued at room temperature for 1 h. Finally, the solution was dried on the sand bath at 70 °C for 20 h and then in the heated oven at 120 °C for 24 h. Sample was calcined at 600 °C for 4 h (catalyst denoted as VHf SG).

### 2.2. Characterization

N_2_ physisorption at −196 °C was performed with Micromeritics ASAP2020 analyzer (Micromeritics ASAP 2020, Norcross, GA, USA) to determine the specific surface areas, pore sizes, and total pore volume distributions of the catalysts. Specific surface areas were calculated with the Brunauer–Emmet–Teller (BET) method and the pore sizes and total pore volume distributions with the Barrett–Joyner–Halenda (BJH) method.

Crystalline structures were determined from X-ray Diffraction patterns (XRD) recorded on PANalytical X’PertPRO diffractometer (PANalytical B.V., Almelo, The Netherlands) equipped with a copper anode (λ = 1.5406 Å). Diffractograms were collected using the step-size of 0.0167° and 2θ range from 15 to 80 with a count time of 100 s per step. The diffraction patterns were identified with the “Joint Committee on Powder Diffraction Standards” (JCPDS) files.

Timegated^®^ 532 Raman Spectrometers (Models: M1 and Pico, Timegate Instruments, Inc., Oulu, Finland) were used to determine the vanadia and sulfur species of the catalysts. The Raman spectra were measured using a fiber coupled pulsed 532 nm laser and a single photon counting CMOS SPAD matrix detector. The data were collected (model Pico Raman) with the Raman shift range from 100 to 2100 cm^−1^ and the spectral resolution of 5 cm^−1^. With model M1, the spectra were collected between 1100–100 cm^−1^ and 1100–2100 cm^−1^ with the spectral resolution of ~10 cm^−1^.

A PANalytical^®^ AXIOS mAX 4 kW X-ray fluorescence (XRF, Malvern, UK) spectrometer was used to determine the elemental compositions of the catalysts. For the analysis, 200 mg of the sample was mixed with 8 g of fusion chemicals (Lithium tetraborate 66%: Lithium metaborate 34%) and melted in an Eagon 2 furnace. The chemical compositions of the catalysts were analyzed with the Omnian standardless method.

The amount of sulfur was quantified from 20 mg of poisoned catalysts using Leco CS-200 analyzer (LECO corporation, Saint Joseph, MI, USA). 0.9 h iron chip accelerator and 1.2 g combustion accelerator were used in analysis. Certified reference materials OREAS 45e (S% = 0.043) and Geostat GCC-07 (S% = 0.51) were used in the calibration of the device.

A scanning transmission electron microscope ((S)TEM) Jeol JEM-F200 (JEOL Ltd., Tokyo, Japan), together with energy dispersive spectrometer (EDS) Jeol Dual EDS for F200 (JEOL Ltd., Tokyo, Japan), was used to study the morphology and crystallography of catalytic materials and distribution of vanadium in them. (S)TEM samples were prepared by crushing a catalyst powder between microscope slides and dispersing the powder with isopropanol onto a holey-carbon-coated copper grid.

### 2.3. Catalyst Activity Studies

The performance of the catalysts in the utilization of methanol (MeOH) and methanethiol (MT) in formaldehyde production were evaluated with light-off tests. MT was selected to represent the reduced sulfur compounds present in pulp mill emissions [21]. Experiments were performed with the laboratory scale equipment presented in ref. [5]. Before experiments, 100 mg of the sample and 900 mg of quartz sand were packed as three separate layers in a tubular quartz reactor. The concentrations of methanol and methanethiol in the mixture were both set to 500 ppm in synthetic air and verified via the reactor by-pass line. The total gas flow was 1 L min^−1^. In the light-off tests, the oven was heated from 100 °C up to 600 °C with the heating rate of 5 °C min^−1^. The composition of the gas flow was measured with an FT-IR analyzer (Gasmet^TM^ CR-2000, Vantaa, Finland). The measured compounds were dimethyl disulfide (C_2_H_6_S_2_), dimethyl sulfide (C_2_H_6_S), methanethiol (CH_3_SH), methanol (CH_3_OH), carbon dioxide (CO_2_), carbon monoxide (CO), formaldehyde (CHOH), formic acid (CH_2_O_2_), methane (CH_4_), nitrogen dioxide (NO_2_), nitrogen monoxide (NO), nitrous oxide (N_2_O), sulfur dioxide (SO_2_), sulfur trioxide (SO_3_), and water vapor (H_2_O).

### 2.4. Poisoning Treatment

Poisoning of the selected catalysts was carried out using SO_2_ and water vapor. The catalyst was placed in a vertically positioned tubular quartz reactor and heated to 400 °C with heating rate of 10 °C min^−1^ under a gas mixture of 10 vol-% air and 90 vol-% N_2_. Five-hour poisoning treatment was performed under the gas mixture of following composition: 100 ppm SO_2_, 10 % H_2_O, 10 % air, and balance N_2_. After the poisoning, the reactor was cooled down for 30 min under a gas mixture of 10 vol-% air and 90 vol-% N_2_. In all the steps, the total gas flow was 1 L min^−1^.

## 3. Results and Discussion

### 3.1. Characterization

The research reported in this paper focuses on the supported vanadia catalysts prepared via impregnation or sol–gel methods. The same vanadia precursor was used in both types of catalysts, while in sol–gel preparation, precursors of hafnia and zirconia were used instead of readily available oxides. Table 1 shows the specific surface areas, porosity information, V_2_O_5_ loadings, and VO_x_ surface densities of the catalysts. Specific surface areas of the samples are quite low and only the specific surface area of 3VHf SG catalyst reaches the value above 10 m^2^g^−1^. The specific surface areas are slightly higher for the sol–gel prepared catalysts. Impregnation of vanadia on ZrO_2_ and HfO_2_ leads to a minor decrease in specific surface area. The low specific surface area results in a high surface density of vanadia (13–96 VO_x_ nm^−2^), which is well above the monolayer coverage of ZrO_2_ and HfO_2_ (monolayer coverage is about 7.7 V nm^−2^ [16]). For this reason, V_2_O_5_ crystals in addition to mono- and polymeric surface VO_4_ species on dehydrated catalyst are expected to exist. The VO_x_ surface density is also calculated [22] for sol–gel prepared samples, even though vanadia species should in this case also be present deeper in the structure.

**Figure 1:**
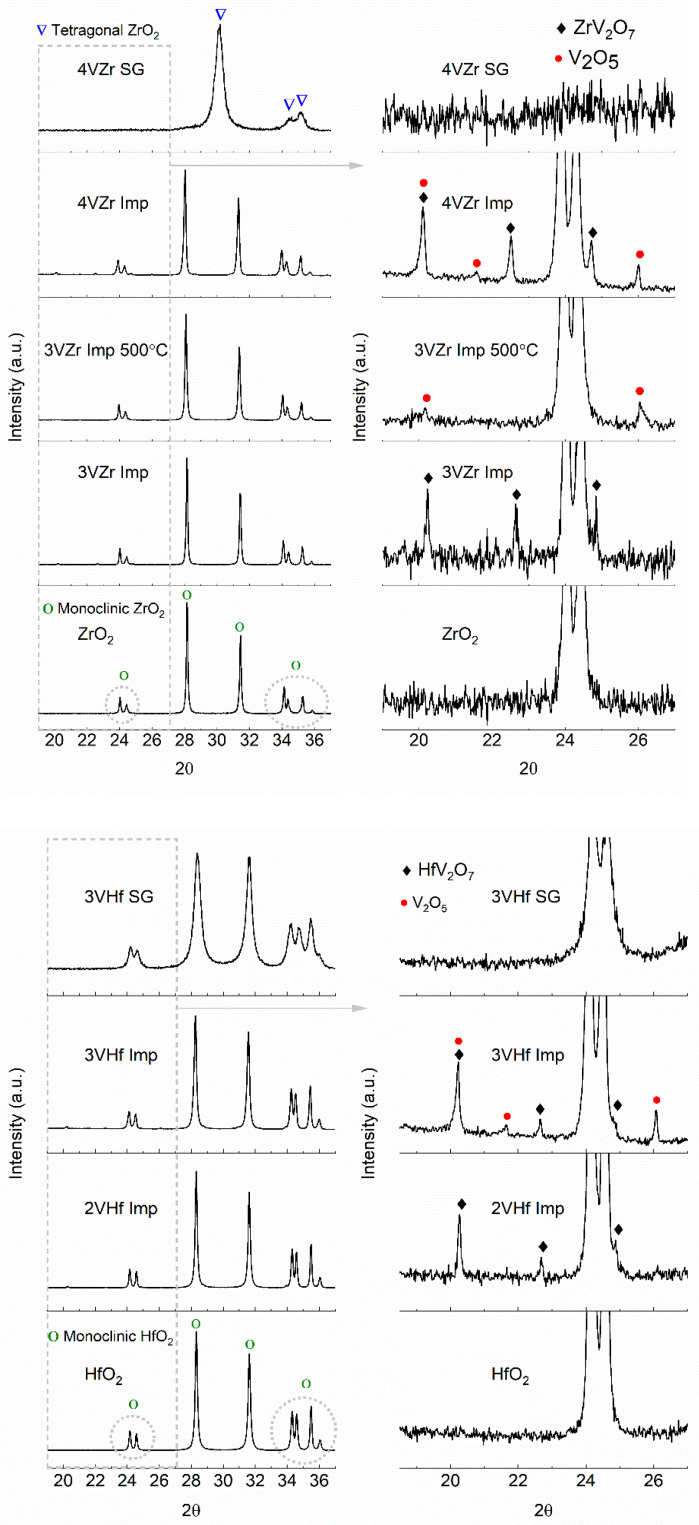
XRD diffractograms for impregnated and sol–gel prepared VZr and VHf catalysts.

XRD analyses show a formation of HfV_2_O_7_ (JCPDF-file: 00-030-0614) and ZrV_2_O_7_ (JCPDF-file: 01-088-0587) phases for impregnated samples calcined at 600 °C (Figure 1). The impregnated 4VZr catalyst contains also V_2_O_5_ in addition to ZrV_2_O_7_, which is not the case with lower-loaded 3VZr imp catalyst. The 3VZr imp catalyst calcined at 500 °C for 2 h seems to contain only crystalline V_2_O_5_ species. No clear indication of co-existence of ZrV_2_O_5_ phase was detected. Monoclinic phase of hafnia (JCPDF-file: 03-065-1142) and zirconia (JCPDF-file: 03-065-1023) were observed for impregnated catalysts.

In the case of sol–gel prepared catalysts, crystalline vanadia species were not observed. The sol–gel preparation of the VZr catalyst leads to formation of mainly tetragonal zirconia phase in addition to smaller amounts of monoclinic zirconia (See also Supplementary Figure S1), while for VHf the same monoclinic hafnia phase is observed for both the catalyst types (impregnated and sol–gel prepared).

Monoclinic phases of ZrO_2_ and HfO_2_ are stable at low temperatures. The tetragonal phase is normally reached upon heating monoclinic ZrO_2_ to 1170 °C and HfO_2_ up to 1600 °C [7,8]. Therefore, changes in zirconia phase between impregnated 3VZr catalyst calcined at 500 °C and 600 °C were not expected. Formation of metal-mixed oxide phases between vanadia and these supports have been earlier observed during oxidation at elevated temperature (above 550 °C), and it has been postulated to have a connection with the phase change of the support [23]. In this case, no phase change of the support was observed based on XRD when calcining the impregnated catalyst at 600 °C, and still the metal-mixed oxide phase was formed. The difference in the results may arise from significantly higher surface vanadia loading of the catalysts in the current study (10-folded value compared to the study of Olthof et al. [23]).

**Figure 2:**
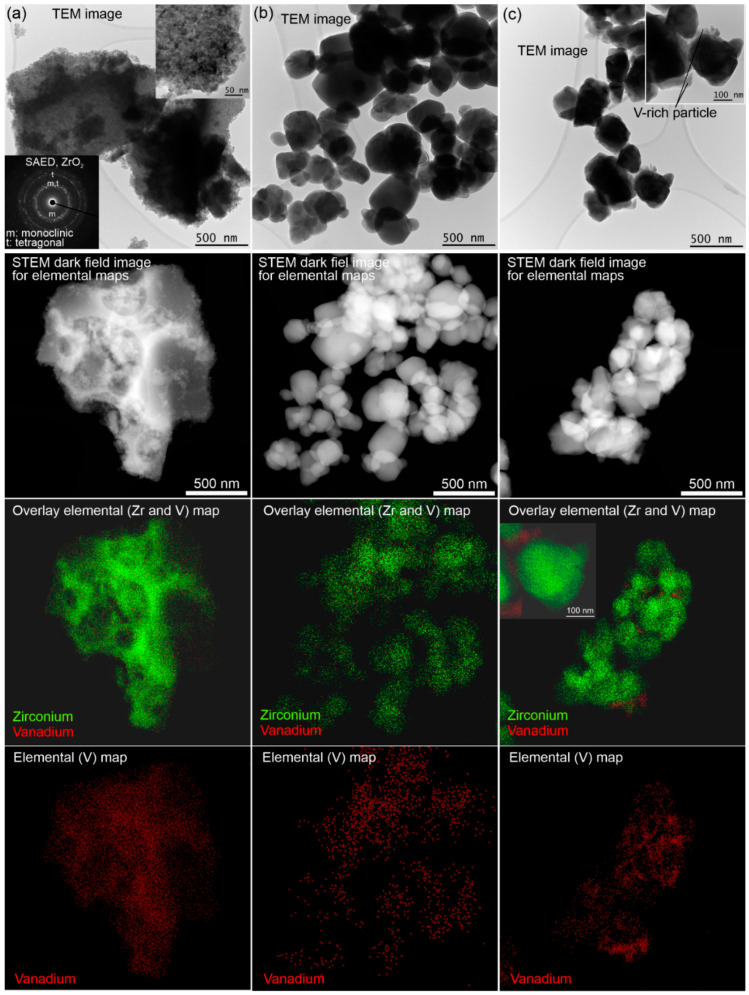
Analytical (S)TEM analysis of VZr catalysts. (**a**) 4VZr SG, (**b**) 3VZr Imp, and (**c**) 3VZr Imp 500 °C.

**Figure 3:**
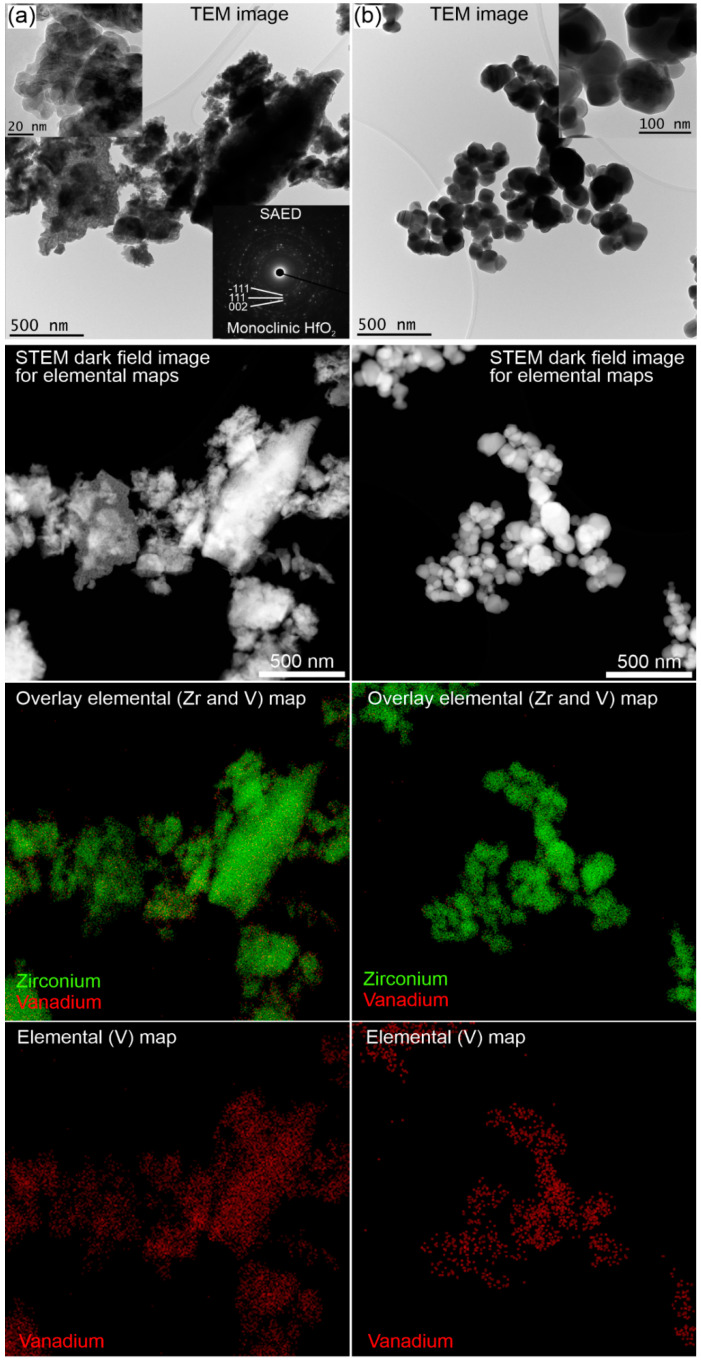
Analytical (S)TEM analysis of VHf catalysts. (**a**) 3VHf SG and (**b**) 2VHf Imp.

Analytical (S)TEM results for sol–gel made and impregnated VZr and VHf catalysts are presented in Figure 2 and Figure 3, respectively. The catalysts were calcined at 600 °C if not otherwise noted. Based on the (S)TEM studies together with selected area electron diffraction (SAED) patterns, the ZrO_2_ support of the sol–gel prepared 4VZr has a tetragonal and monoclinic structure with a particle size <50 nm (Figure 2a). The HfO_2_ support of the sol–gel prepared 3VHf has a monoclinic structure with a particle size <20 nm (Figure 3a). These results agree well with XRD results (Figure 1). The impregnated 3VZr and 2VHf catalysts (Figure 2b,c and Figure 3b) have a particle size of mainly >100 nm and >50 nm, respectively. Because of the thick particles, collecting clear SAED patterns was challenging, however, monoclinic ZrO_2_ and monoclinic HfO_2_ phases could be verified from them agreeing with the XRD results (Figure 1). The STEM-EDS analyses (Figure 2b and Figure 3b) indicate that vanadium in the sol–gel prepared and impregnated (calcined at 600 °C) samples, both VZr and VHf, is well-distributed. In the case of the impregnated catalyst calcined at 500 °C (3VZr; Figure 2c), crystalline, plate-like vanadium-rich particles between ZrO_2_ particles were observed, being most probably V_2_O_5_ particles based on the XRD results (Figure 1). Longer calcination carried out at a higher temperature seems to produce better vanadium distribution. Normally, one would expect that vanadia surface layers are formed before V_2_O_5_ species due to the differences of surface free energy of oxygen terminated vanadia and OH -terminated support oxide, and the high mobility of V_2_O_5_ due to its low melting point [16]. It seems that high surface VO_x_ concentration and lower calcination temperature than the V_2_O_5_ melting point leads to nonhomogeneous vanadia distribution.

**Figure 4:**
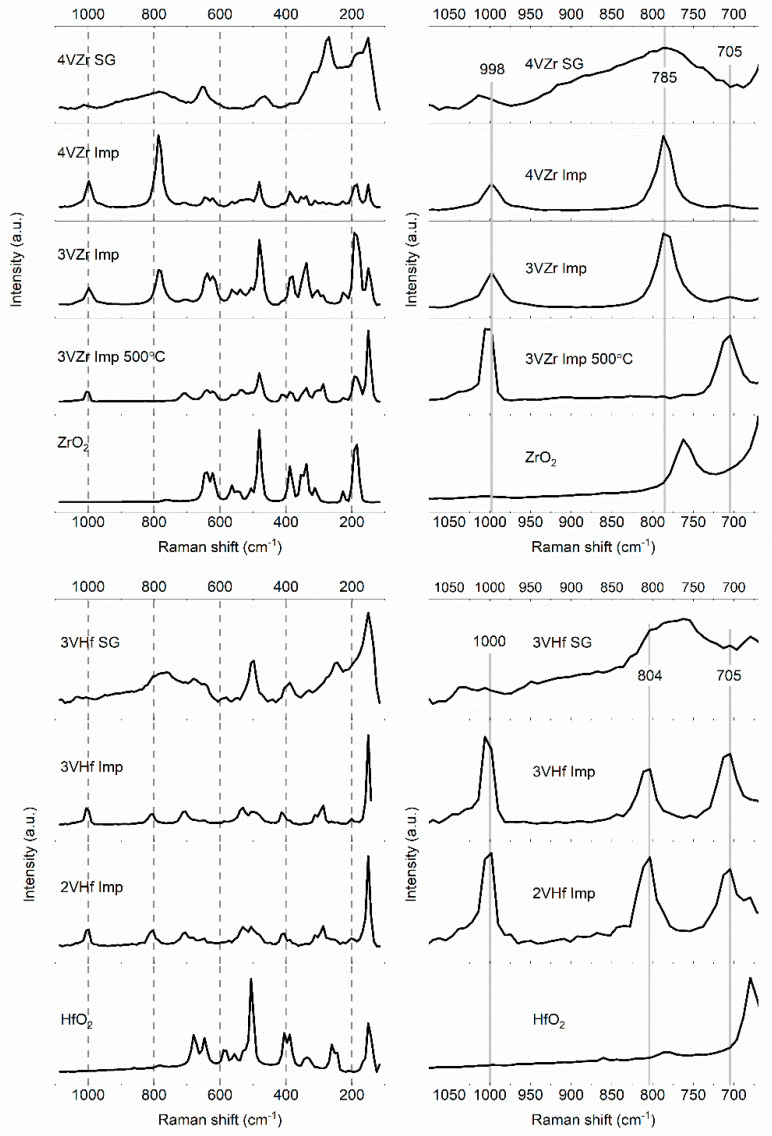
Raman spectra of ZrO_2_, HfO_2_, and prepared catalysts.

Raman spectroscopy is immensely powerful tool in studies related to vanadia catalysts. Even though highly crystalline materials give stronger peaks in the spectra, also information on less structured materials can be achieved revealing the species of vanadia from sol–gel prepared catalysts in this case. The results of Raman-analysis (Figure 4) give indications related to the tetragonal phase of ZrO_2_ in the sol–gel prepared sample, although due to low intensity of the signals, co-existence of monoclinic phase is possible [24,25]. In other samples, ZrO_2_ and HfO_2_ are in monoclinic form [24,26,27]. These results are consistent with the XRD and TEM analysis.

As mentioned, V_2_O_5_ crystals are expected to appear along with other possible vanadia species in these materials. The peak observed at ~1000 cm^−1^ is an indication of V_2_O_5_ crystals, which is supported by the peak observed at around 150 cm^−1^. V_2_O_5_ crystals are present on an impregnated VZr catalyst that was calcined at 500 °C. The presence of V_2_O_5_ was observed also in the XRD and STEM-EDS analyses. Longer calcination (4 h) at 500 °C does not change the structure of the vanadia species markedly, even though the amount of V_2_O_5_ particles could be quantitatively somewhat higher (See comparison in supplementary material Figure S2). Increasing the calcination temperature near the melting point of vanadium pentoxide, an intense peak appears at 785 cm^−1^ due to formation of ZrV_2_O_7_. Another characteristic peak of ZrV_2_O_7_ is typically observed at around 980 cm^−1^. In the case of 4VZr catalyst, the V_2_O_5_ peak is widened, and a shoulder appears at around 980 cm^−1^. The observed vanadia phase change is also involved with a decrease in the specific surface area of the catalyst. In contrast to what was found by Olthof et al. [23], phase change of zirconia was not observed in connection with metal-mixed oxide formation. An interesting new peak at 705 cm^−1^ is noticed for impregnated VZr sample that was calcined at 500 °C. The peak appears more intense for the sample that was calcined during 4 h. This unusual Raman signal has earlier been observed to appear in the spectra of thin zirconia layers grown on zirconium alloys. Ciszak et al. [28] explained the peak as a band that describes disorder or defects in the material. The band is normally symmetry-forbidden, but it becomes visible with a loss of symmetry in the material. This may explain how vanadium is inserted in zirconia structure while no apparent zirconia phase change is observed. The band at 705 cm^−1^ is also visible in impregnated VZr catalysts calcined at 600 °C, where the formation of ZrV_2_O_5_ is clearly evidenced.

In the case of sol–gel prepared VZr catalysts, the peak indicating vanadia species is observed at around 1020 cm^−1^. This is related to V = O stretching of monovanadate species [23]. The broad band from about 700 to 980 cm^−1^ is related to polyvanadate species containing numbers of V-O-V and V-O-support bonds, which makes the observed spectral feature broad [29]. The sol–gel prepared materials are less crystalline, shown by wider and less intense Raman signals than that for the impregnated catalysts. For this reason, one cannot observe a clear peak at 785 cm^−1^, indicating ZrV_2_O_7_ phase, but due to overlapping of polyvanadate modes we cannot exclude its possible presence [23].

Due to chemical similarity of zirconia and hafnia, equivalent Raman results are observed for VHf catalysts. The peaks appearing at around 1000 cm^−1^ and 800 cm^−1^ for impregnated VHf catalysts are indicative for the formation of HfV_2_O_7_. Since the higher wavenumber peak is at somewhat higher frequency than expected, co-existence of V_2_O_5_ crystals is possible. Furthermore, additional peak indicating disorder in the case of VZr catalysts is also observed for VHf. The “disorder” peak at 705 cm^−1^ for impregnated 3VHf and 2VHf calcined at 600 °C is relatively more intense than in the case of impregnated 3VZr. For the sol–gel prepared VHf catalyst, the results are similar than for VZr catalyst [23].

### 3.2. Production of Formaldehyde from Methanol and Methanethiol Mixture

The reactions of methanol (oxidative dehydrogenation) and methanethiol (oxidative desulfurization) to formaldehyde can be described by the following equations [5,30,31]:

CH_3_OH + ½ O_2_ -> HCHO + H_2_O (1)

CH_3_SH + 2O_2_ -> HCHO + SO_2_ + H_2_O (2)

The main products of both the reactions are formaldehyde and water. Sulfur in methanethiol molecule is oxidized to SO_2_. In excess oxygen, formaldehyde can react further according to reactions [5,31]:

HCHO + ½ O_2_ -> CO + H_2_O (3)

HCHO + O_2_ -> CO_2_ + H_2_O (4)

Methanethiol can also react to dimethyl disulfide (DMDS) (Equation (5)) and dimethyl sulfide (DMS) (Equation (6)) [5]:

2CH_3_SH + ½ O_2_ -> CH_3_SSCH_3_ + H_2_O (5)

2CH_3_SH + 1 ½ O_2_ -> CH_3_SCH_3_ + H_2_O + SO_2_ (6)

DMDS and DMS can be further reacted according to the Equations (7)–(10) [4,32]:

H_3_SSCH_3_ + O_2_ -> 2HCHO + 2SO_2_ + H_2_O (7)

CH_3_SCH_3_ + 2 ½ O_2_ -> 2HCHO + SO_2_ + H_2_O (8)

CH_3_SSCH_3_ + 5 ½ O_2_ -> 2CO_2_ + 2SO_2_ + 3H_2_O (9)

CH_3_SCH_3_ + 4 ½ O_2_ -> 2CO_2_ + SO_2_ + 3H_2_O (10)

**Figure 5:**
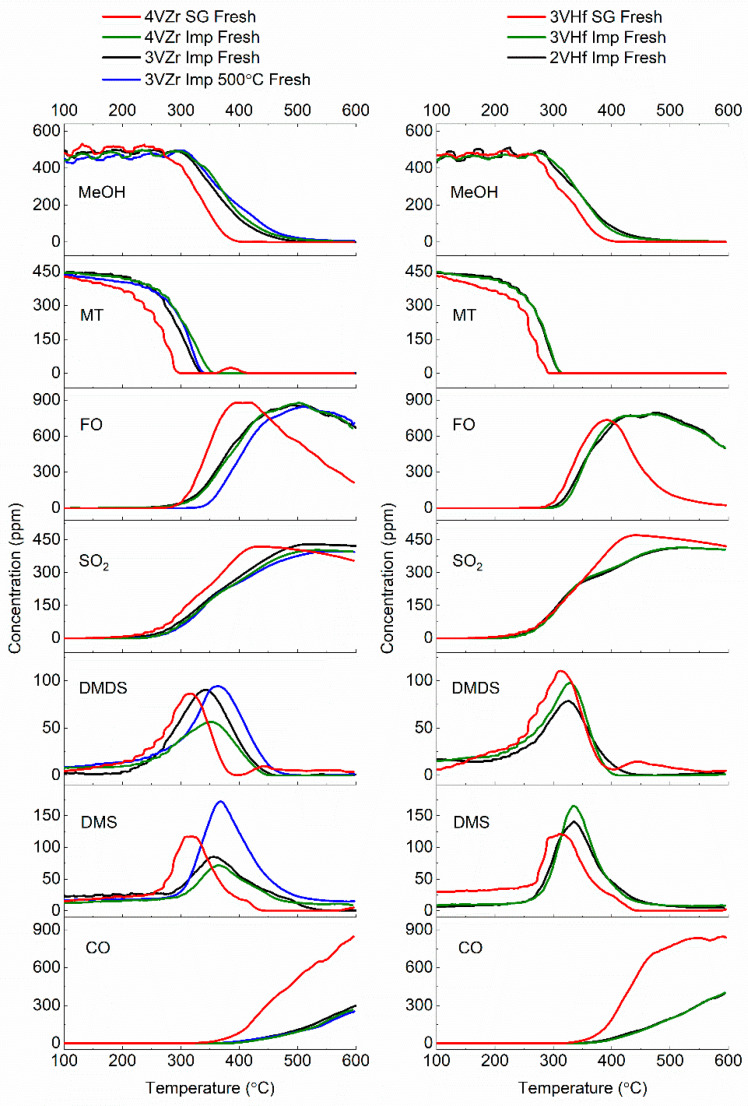
Formaldehyde (FO) production from methanol (MeOH) and methanethiol (MT) over the studied fresh catalysts; consumption of reactants and formation of different reaction products.

The results of the experiments realized with VZr and VHf catalysts (Figure 5) show that methanethiol reaction starts at a lower temperature range than that of methanol. This is due to the lower total dissociation energy and longer C–S bond of methanethiol compared with the bonds of methanol molecule [5,33]. The catalysts prepared using sol–gel method reach the maximum formaldehyde concentration at a significantly lower temperature range (~400 °C) than the impregnated catalysts (~500 °C). However, the reaction of formaldehyde towards CO over sol–gel catalysts begins rather soon after reaching the maximum formaldehyde concentration, while impregnated catalysts can keep formaldehyde production until close to ~600 °C. The impregnated VZr catalyst that was calcined at 600 °C was slightly better compared to the one calcined at 500 °C, which was visible, for example, in formaldehyde production. The maximum theoretical selectivity of formaldehyde from the reaction mixture is 67% [6]. In this case, the selectivities are close to maximum, reaching 60–63%. The formaldehyde selectivity in the case of 3VHf SG catalyst was slightly lower, being 57% at maximum (See more information in Supplementary Material, Table S1).

The concentrations of dimethyl disulfide (DMDS) and dimethyl sulfide (DMS) reach the maximum at the same temperature level where methanethiol conversion is complete. The SO_2_ formation finds the maximum when DMDS and DMS are completely consumed. This is also the same temperature level where maximum formaldehyde production is achieved. This temperature level would be optimal for the formaldehyde production, since then the reaction intermediates are consumed, but further reaction of formaldehyde towards CO and CO_2_ is still at a low level. The catalytic materials prepared via sol–gel and impregnation methods produce similar reaction intermediates, however, slightly lower intermediate production amounts for impregnated catalysts can be noted. Calcination of impregnated ZVr catalyst at 600 °C shifts the production of intermediates at somewhat higher temperature level. Additionally, the amounts are affected, showing decreased formation, especially of DMS.

The amount of vanadium (3 vs. 4 wt.%) does not have a remarkable impact on methanol and methanethiol conversions or formaldehyde yield, as demonstrated by impregnated VZr catalysts. Both these catalysts have ZrV_2_O_7_ species, however in different amounts. Furthermore, 4VZr Imp catalyst contains more V_2_O_5_ species than the 3VZr Imp catalyst, since V_2_O_5_ was not observed in XRD analysis of 3VZr imp, even though it is known to be present in the catalyst based on Raman analysis. It has been earlier noted that V_2_O_5_ species is not equally active to, for example, monomeric and polymeric vanadia species in methanol oxidative dehydrogenation. When V_2_O_5_ nanoparticles start to form, the activity of catalyst is observed to decrease due to build-up of nanoparticles on more active vanadia sites [16]. The slightly better conversions of methanol and methanethiol in case of 3VZr Imp could be an indication of decrease in activity when V_2_O_5_ is formed in higher amounts in 4VZr Imp. However, due to a very small difference, this should be confirmed with a catalyst having even higher loading of vanadia. Increase in ZrV_2_O_7_ amount seems not to increase the activity markedly. However, comparison of the reactant consumptions and formaldehyde production of 3VZr Imp calcined at 600 °C with 3 VZr imp calcined at 500 °C, the slightly better activity of ZrV_2_O_7_ species compared to V_2_O_5_ can be suggested. Different vanadia loadings lead to differences in DMDS and DMS production. More DMDS is formed over 3VZr Imp catalyst. A higher amount of V_2_O_5_ on the 4VZr Imp catalyst cannot explain the lower by-product formation, since the 3VZr Imp 500 °C catalyst containing only V_2_O_5_ species shows equal formation of DMDS than the 3VZr Imp catalyst. It seems that lower DMDS formation with 4VZr Imp is related to the higher amount of ZrV_2_O_7_ species. Identical conclusions can be made related to the impregnated VHf catalysts having different loadings of vanadia (See Figure 4). In this case, methanol or methanethiol conversions are very similar, although V_2_O_5_ species are observed in XRD analysis of 3VHf Imp, while for 2VHf Imp the V_2_O_5_ species is visible only in Raman.

The sol–gel prepared catalysts in both cases were more active than the impregnated counterparts. The vanadia species on the sol–gel prepared catalysts took mono- and poly-vanadate structures, and principally no crystalline vanadia species were observed. The existence of HfV_2_O_7_ and ZrV_2_O_7_ could not be ruled out based on Raman analysis, however based on the results discovered with impregnated catalysts, the metal-mixed oxide phase is expected to have similar or only slightly higher activity than V_2_O_5_. Based on the results, the mono- or polymeric vanadia species seem to be more active species than the others observed in production of formaldehyde from mixture of methanol and methanethiol.

The vanadia species are known to have three differently bonded oxygen atoms: V = O, V-O-support bonding, and bridging V–O–V. It has been earlier demonstrated that methanol adsorption occurs via dissociative chemisorption as surface methoxy species and hydrogen at the bridging V-O-support bond. Hydrogen atom forms surface hydroxyl with the oxygen atom and methoxy species is coordinated to the vanadium site. Subsequently, V–OCH_3_ decomposes to formaldehyde and water. It has also been discovered that the mechanism follows Mars-van Krevelen kinetics due to independence of the surface kinetics on the gas phase molecular oxygen. Earlier steady-state kinetic studies have also shown that methanol reaction to formaldehyde proceeds on one surface VO_4_ site when vanadia coverage is less than monolayer. The specific activities of monomeric and polymeric vanadia species are equivalent. Above the monolayer, the reaction rate is decreased due to formation of V_2_O_5_ particles. Methanol reaction is also very sensitive to the support since V-O-support bond is related to the rate-determining step of the methanol reaction. Lower electronegativity of the support cation improves the bond’s redox activity and low oxygen defect formation enthalpy of supported vanadia sites will lead to higher reaction rates [16]. HfO_2_ and ZrO_2_ are chemically very similar, and ZrO_2_ cation electronegativity is only slightly higher (1.33 for Zr and 1.3 for Hf (Pauling scale)) [34]. Based on Raman analysis, HfO_2_-supported impregnated catalysts contain more defect sites than the corresponding ZrO_2_-supported catalysts. The activity results support these earlier findings, since 3VHf Imp catalyst is slightly better than corresponding 3VZr Imp catalyst (See Supplementary Material, Figure S3). To further discover the effect of sulfur on the performance of these catalysts, a poisoning treatment under SO_2_ and water vapor was done.

**Figure 6:**
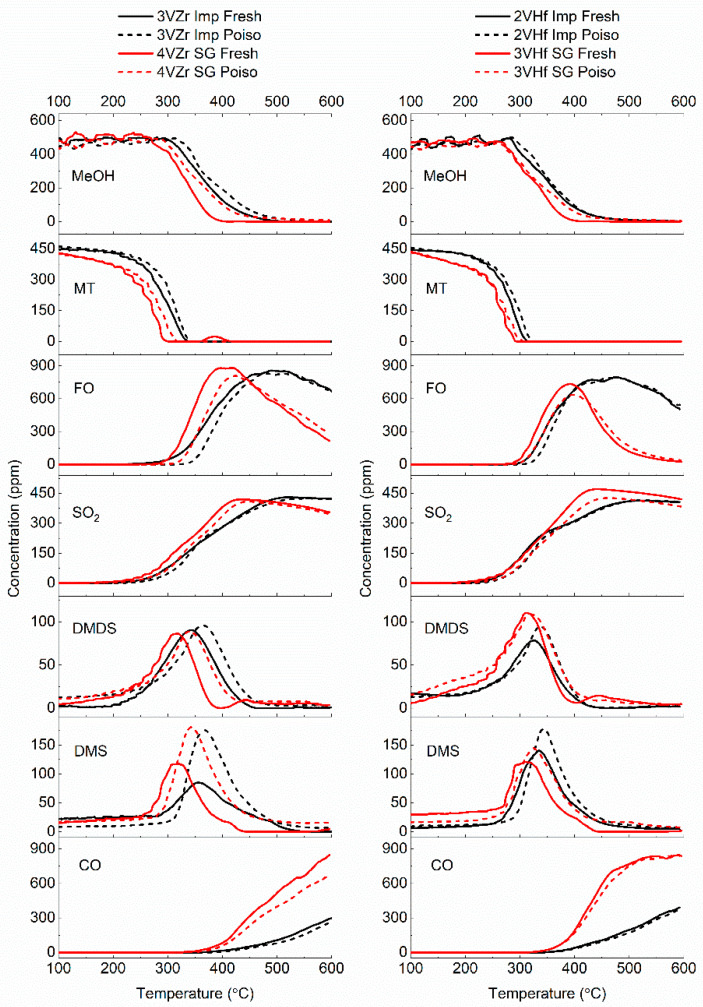
Comparison of fresh and poisoned catalysts; consumption of reactants and formation of different reaction products.

Comparison of poisoned catalysts and the fresh counterparts (Figure 6) demonstrates that the impregnated catalysts were slightly more stable. Formaldehyde and SO_2_ production of the sol–gel catalysts were especially decreased. It can be postulated that mono- or polymeric vanadia species are more sensitive to the poisoning treatment than V_2_O_5_ and metal-mixed oxide species observed in impregnated catalysts. Dunn et al. [35] have earlier studied oxidation of sulfur dioxide over several metal oxide catalysts and found that the bridging V-O-support bond is responsible on SO_2_ adsorption and following oxidation. The similar conclusions in the case of SO_2_ can be made concerning the electronegativity of the support cation and the activity of the V-O-support site than earlier in methanol reaction [35]. This explains why sol–gel prepared catalyst was more affected by the poisoning treatment and why HfO_2_ supported catalysts are slightly less affected than the ZrO_2_ supported catalysts. DMS formation seems to increase after poisoning, which is probably due to the reaction of sulfur from the catalyst surface. A similar increase in DMDS formation cannot be observed. As we know that DMDS is formed easily from methanethiol—even without a catalyst—and that DMS is always formed at a slightly higher temperature than DMDS, we can hypothesize that DMS formation is involved with presence of specific sulfur species on the catalyst surface. Increasing amount of DMS formation after poisoning the catalyst supports this hypothesis. The same observation related to the by-products is valid for both VZr and VHf, independent on the preparation method used. Therefore, it can be hypothesized that higher formation of DMS is related to vanadia species that exist in all the types of catalysts under the study, or it is a property of the support. In general, the differences in catalyst performances after poisoning were quite small, indicating the rather good tolerance of all the catalysts against sulfur poisoning.

The sulfur amount in poisoned samples was measured using C-S analyzer. The poisoning was done at 400 °C that was selected based on maximum formaldehyde production temperature. The results showed (See Table 2) that catalysts prepared using sol–gel method are retaining higher amounts of sulfur compared to the impregnated catalysts, although being rather low in general. This may have a connection to the mono- and polymeric vanadia species on the sol–gel-prepared catalysts, as the V-O-support site is known to have an important role in both SO_2_ and methanol reactions. The activity experiments after the poisoning revealed that sol–gel prepared catalysts were slightly more affected by the poisoning treatment than the impregnated ones, which is in line with the amounts of sulfur detected.

**Figure 7:**
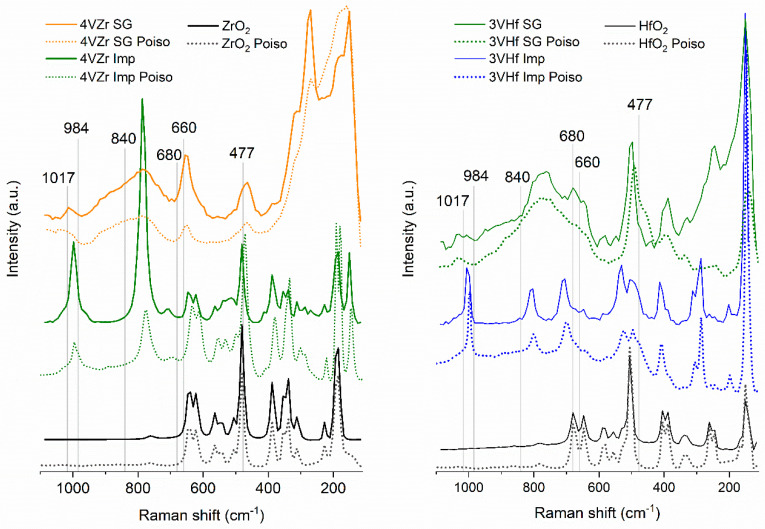
Raman spectra of the poisoned catalysts, HfO_2_ and ZrO_2_, together with corresponding fresh materials.

Raman spectra of the poisoned 4VZr Imp, 4VZr SG, 3VHf Imp, 3VHf SG catalysts, HfO_2_, and ZrO_2_ are presented in Figure 7. The spectra of the other catalysts are presented in Supplementary Figures S4 and S5. In general, the spectra of the impregnated catalysts seem to be less affected by the poisoning treatment as expected based on earlier results. In the case of impregnated 4VZr sample, the spectral features of the support become more intense in comparison to vanadia species. This could be an indication of covering or interaction of V_2_O_5_ and ZrV_2_O_7_ species with sulfur. Due to lower activity of these species in the reaction in concern, the observed decrease in performance is not substantial. The S-O stretching mode normally appears at around 1020 cm^−1^ [25], and it is not observed in the case of impregnated 4VZr catalyst. The widening of the spectral feature in case of sol–gel prepared 4VZr catalyst could indicate presence of S-O stretching mode of the sulfate. The Raman spectra of poisoned ZrO_2_ and HfO_2_ supports show appearance of small band at around 1025 cm^−1^ (See Supplementary Figures S6 and S7). This could be an indication of S-O stretching mode, since the spectral feature is not affected by the presence of vanadium species in the same region. The Raman spectra measured in the range of 1100–2100 cm^−1^ for poisoned impregnated 3VHf, 3VZr, and 4VZr catalysts (presented in Supplementary Figure S8) show weak vibrations at around 1380–1400 cm^−1^ for 4VZr Imp catalyst, which may be related to the presence of S = O bond stretching of the sulfate [25]. Changes in V-O-support region of sol–gel prepared catalysts point to interference of sulfur in poisoned 3VZr SG and 3VHf SG catalysts. (Note that the differences are not visible in Figure 7 due to modified y-axes for clearer presentation; non-adjusted spectra are presented in Supplementary Figures S9 and S10.) Vanadia peaks of 3VHf SG catalyst seem to be less impacted by the poisoning treatment than those of the 4VZr SG catalyst. However, shoulders appear at around 475–465 cm^−1^ in the spectra of poisoned VHf SG catalysts, which may indicate presence of V-O-S vibration [36].

## 4. Conclusions

This work aimed at development of active and stable catalytic materials for the utilization of sulfur contaminated methanol in formaldehyde production. Based on earlier results, rather low surface area hafnia and zirconia were selected as the supports to reach high surface loading of VO_x_ (V nm^−1^). The catalysts were prepared via impregnation and sol–gel methods.

The results showed that:

- vanadia species of dehydrated catalysts differ from each other depending on calcination temperature and preparation method. Impregnation leads to formation of V_2_O_5_ at calcination temperature of 500 °C. At higher calcination temperature (600 °C), vanadia is integrated in the support oxide structure, forming either ZrV_2_O_7_ or HfV_2_O_7_ depending on the support used. At higher vanadia loading, V_2_O_5_ may co-exist with metal mixed oxide structure.
- The major changes in the vanadia species of impregnated catalysts were solely dependent on the calcination temperature and no phase change of the bulk of the support was observed based on XRD and (S)TEM analysis. Raman analysis was able to reveal the formation of support defect sites that could help in formation of crystalline metal-mixed oxide phases.
- The sol–gel preparation of corresponding catalysts leads to formation of mono- and polymeric vanadia species. The hafnia takes a monoclinic structure, while zirconia also contains tetragonal phase in the sol–gel prepared sample. XRD and Raman analyses did not clearly evidence the presence of metal-mixed oxide structure in the case of sol–gel prepared catalysts.
- Impregnation of vanadia leads to a poorer distribution of vanadium on the support, which is especially visible in the catalyst calcined at 500 °C. Poorer distribution increases vanadia surface concentration locally and crystalline V_2_O_5_ is formed. A higher calcination temperature (600 °C) which is closer to vanadia melting point helps in dispersing vanadia more effectively, and metal-mixed oxide phases are formed. The reaction experiments showed the slightly higher activity of the sol–gel prepared catalysts, which arise from the presence of mono- and polymeric vanadia species.
- The results did not indicate higher activity of crystalline metal-mixed oxide phases compared with amorphous mono- and polymeric vanadia phases.
- While all the catalysts were rather stable towards sulfur poisoning, the sol–gel prepared samples retained a higher amount of sulfur, and their performances were decreased slightly after the poisoning treatment. It seems that more active mono- and polymeric vanadia species are more susceptible for the poisoning than V_2_O_5_ and metal-mixed oxide species, due to competing reactions of methanol and SO_2_ on the same active V-O-support site.

## Figures and Tables

**Table 1:** Characteristics of the catalysts and supports, including vanadium amount from XRF analysis calculated as V_2_O_5_, and calculated VO_x_ surface densities. The samples were calcined at 600 °C if not indicated otherwise.

Sample	Specific Surface Area (m^2^g^−1^)	Total Pore Volume (cm^3^g^−1^)	Average Pore Size (nm)	V_2_O_5_ Amount (wt-%)	VO_x_ Surface Density (V nm^−2^)
ZrO_2_	5.0	0.019	15.6	-	-
4VZr SG	5.2	0.013	9.6	4.2	55
4VZr Imp	2.9	0.015	20.5	4.2	96
3VZr Imp	2.9	0.016	21.9	2.8	66
3VZr Imp 500 °C	4.7	0.025	21.0	2.8	39
HfO_2_	4.9	0.034	27.8	-	-
3VHf SG	16.6	0.034	8.3	3.2	13
3VHf Imp	5.2	0.036	27.2	3.5	45
2VHf Imp	4.6	0.026	22.3	2.0	24

**Table 2:** Sulfur amount in poisoned catalysts.

Poisoned Sample (SO_2_ + H_2_O at 400 °C)	Amount of Sulfur (wt.%)
ZrO_2_	0.05
4VZr SG	0.23
4VZr Imp	0.02
3VZr Imp	0.03
3VZr Imp 500 °C	0.02
HfO_2_	0.05
3VHf SG	0.10
3VHf Imp	0.03
2VHf Imp	0.02

## Data Availability

Not applicable.

## Supplementary Materials

The following are available online at https://www.mdpi.com/article/10.3390/ma14185265/s1, Figure S1: XRD diffractogram of 4VZr SG catalyst with 2θ range of 27–54°, Figure S2: Comparison of Raman spectra of impregnated 3VZr catalysts having different calcination treatments, Figure S3: Comparison of 3VZr and 3VHf catalysts; consumptions of reactants and formation of different reaction products, Figure S4: Raman spectra of fresh and poisoned VZr catalysts, Figure S5: Raman spectra of fresh and poisoned VHf catalysts, Figure S6: Raman spectrum of fresh and poisoned HfO_2_ support in the spectral range of 700–1100 cm^−1^, Figure S7: Raman spectrum of fresh and poisoned ZrO_2_ support in the spectral range of 700–1100 cm^−1^, Figure S8: Raman spectra of poisoned 3VHf, 3VZr and 4VZr catalysts in Raman shift range of 1100–2100 cm^−1^, Figure S9: Raman spectra of fresh and poisoned 3VHf SG catalyst. Y-axis is similar for both the spectra, Figure S10: Raman spectra of fresh and poisoned 4VZr SG catalyst. Y-axis is similar for both the spectra and Table S1: Selectivity of formaldehyde at indicated reaction temperature. Temperatures are given with 5 °C accuracy and selectivities given with 1% accuracy.
