# Supplementary material for: Vanadia–Zirconia and Vanadia–Hafnia Catalysts for Utilization of Volatile Organic Compound Emissions
Source: Materials (Basel). 2021 Sep 13;14(18):5265. doi: 10.3390/ma14185265 (PMC8467847; doi:10.3390/ma14185265)
Supplement: Supplementary file 1 [file materials-14-05265-s001.zip › materials-1374623-supplementary.pdf]

materials

Supplementary Materials

Vanadia-Zirconia and Vanadia-Hafnia Catalysts for Utilization of Volatile Organic Compound Emissions

Satu Ojala [1,*], Tiina Laitinen [1], Sian Leneuf de Neufville [2], Mari Honkanen [3], Minnamari Vippola [3], Mika Huuhtanen [1] and Riitta L. Keiski [1]

[1] Environmental and Chemical Engineering, Faculty of Technology, University of Oulu, Finland; firstname.lastname@oulu.fi
[2] Institut Universitaire de Technologie de Poitiers, Universite de Poitiers, France; sianleneuf@gmail.com
[3] Tampere Microscopy Center, Tampere University, Finland; firstname.lastname@tuni.fi
* Correspondence: satu.ojala@oulu.fi;

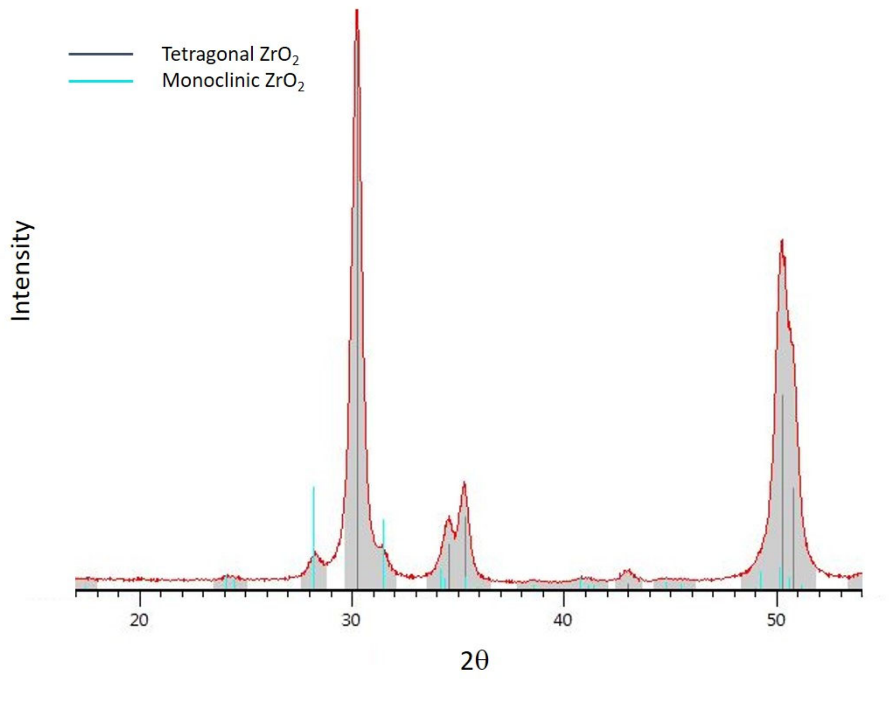

Figure S1: XRD diffractogram of 4VZr SG catalyst with 2θ range of 27–54°.

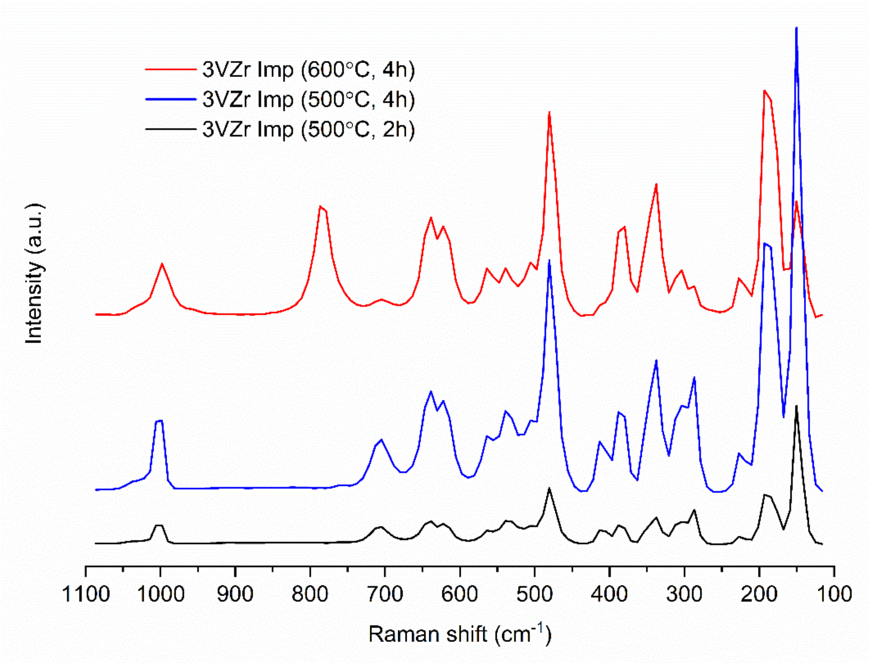

Figure S2: Comparison of Raman spectra of impregnated 3VZr catalysts having different calcination treatments.

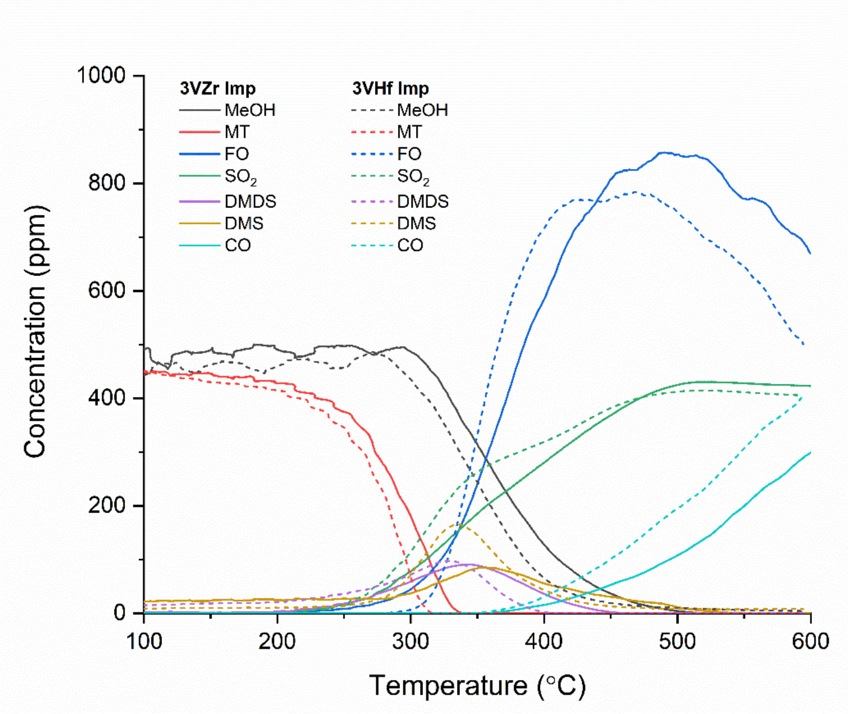

Figure S3: Comparison of 3VZr and 3VHf catalysts; consumptions of reactants and formation of different reaction products.

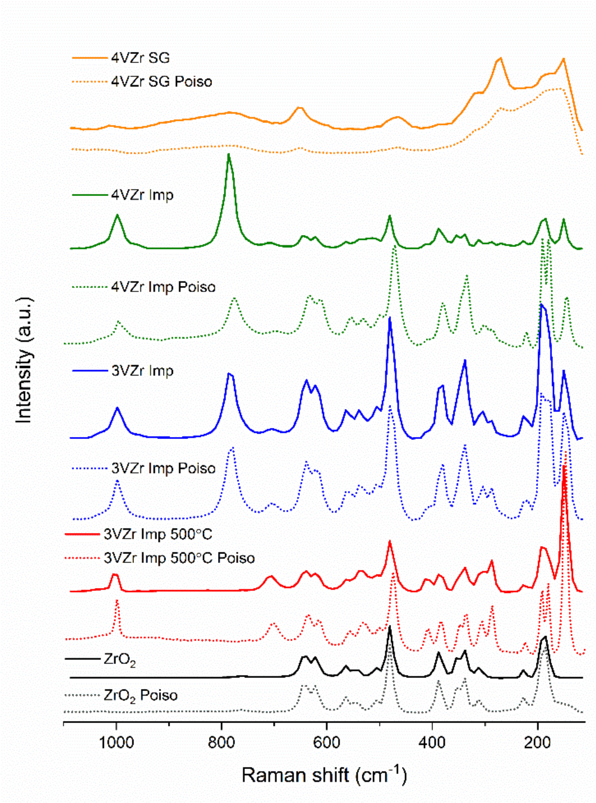

Figure S4: Raman spectra of fresh and poisoned VZr catalysts.

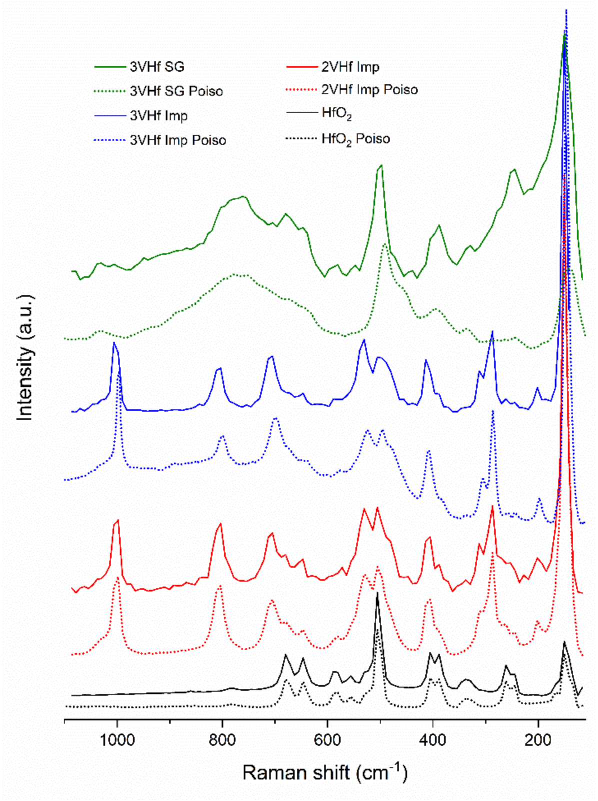

Figure S5: Raman spectra of fresh and poisoned VHf catalysts.

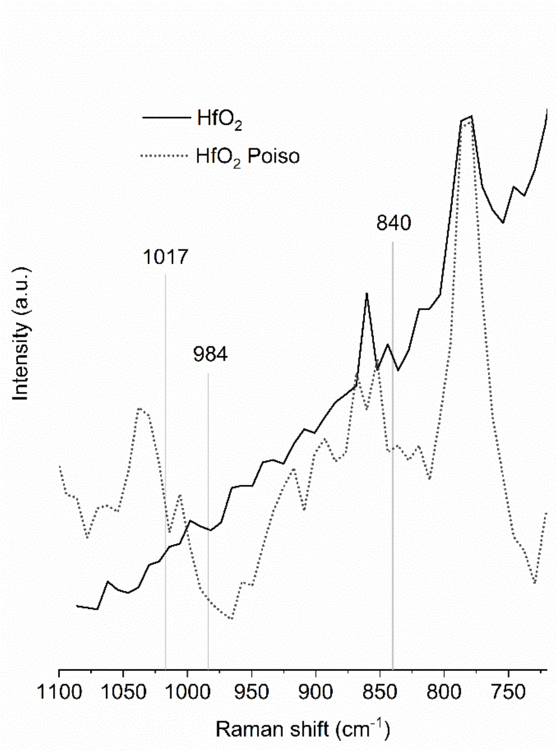

Figure S6: Raman spectrum of fresh and poisoned HfO_2 support in the spectral range of 700–1100 cm^{-1}.

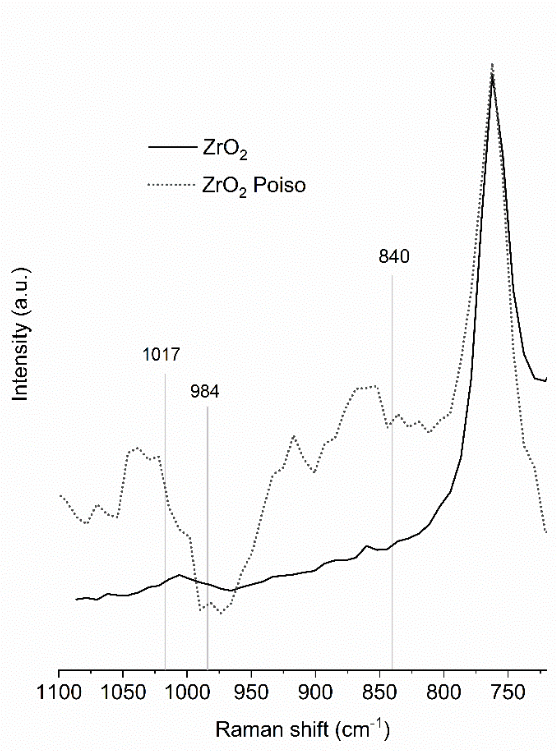

Figure S7: Raman spectrum of fresh and poisoned ZrO_2 support in the spectral range of 700–1100 cm^{-1}.

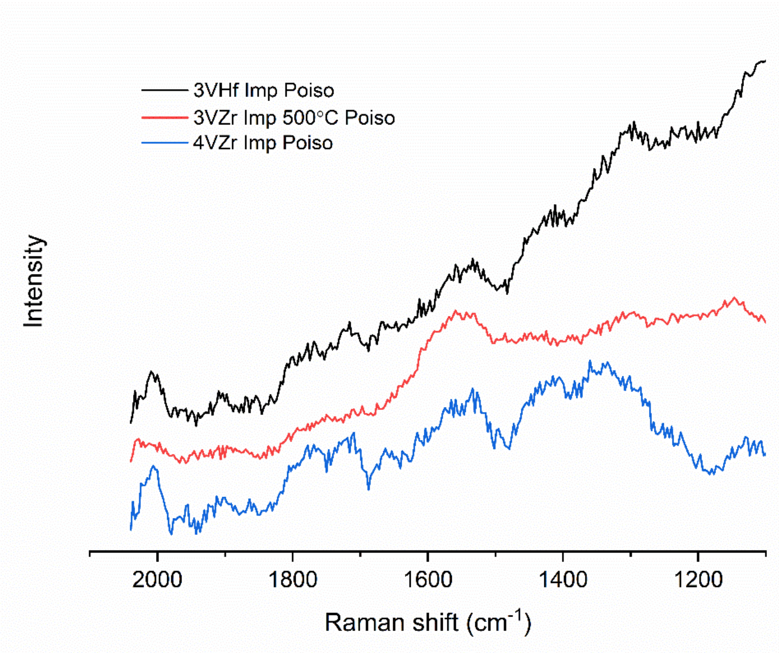

Figure S8: Raman spectra of poisoned 3VHf, 3VZr and 4VZr catalysts in Raman shift range of 1100–2100 cm^{-1}.

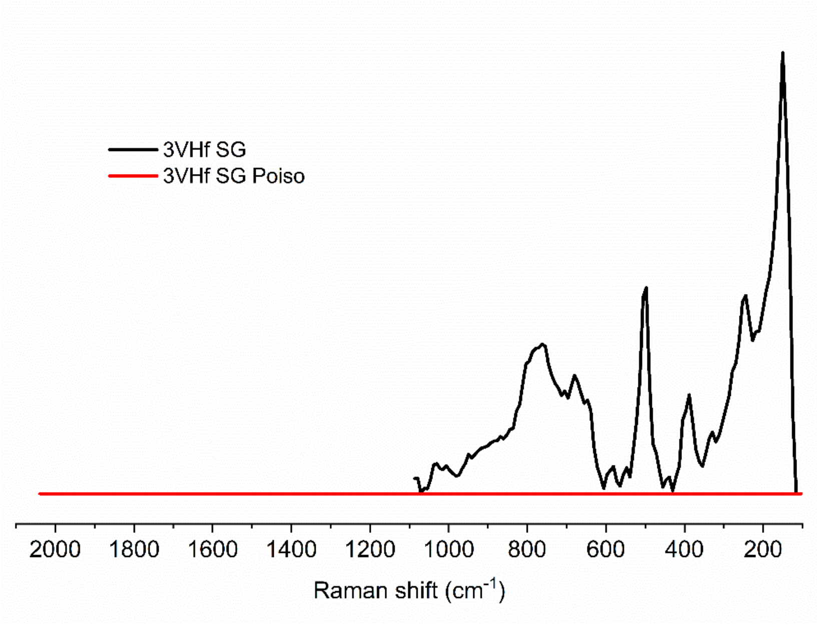

Figure S9: Raman spectra of fresh and poisoned 3VHf SG catalyst. Y-axis is similar for both the spectra.

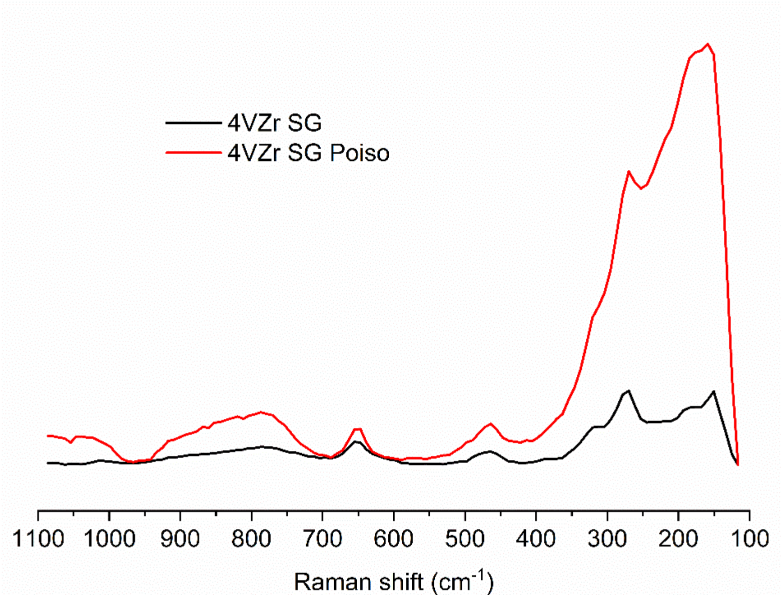

Figure S10: Raman spectra of fresh and poisoned 4VZr SG catalyst. Y-axis is similar for both the spectra.

Table S1: Selectivity of formaldehyde at indicated reaction temperature. Temperatures are given with 5 °C accuracy and selectivities given with 1% accuracy.

**Catalyst**	**Temperature at Maximum Selectivity**	**Maximum Selectivity**	**Temperature at Maximum Formaldehyde Production**	**Selectivity at Maximum Formaldehyde Production**
4VZr SG	375	63	395	63
4VZr Imp	445	63	495	63
3VZr Imp	450	62	485	60
3VZr Imp 500 °C	475	61	505	62
3VHf SG	370	57	390	55
3VHf Imp	405	62	465	58
2VHf Imp	415	60	470	59
